# Supplementary material for: Physiological and Molecular Responses of Vitis vinifera cv. Tempranillo Affected by Esca Disease
Source: Antioxidants (Basel). 2022 Aug 30;11(9):1720. doi: 10.3390/antiox11091720 (PMC9495647; doi:10.3390/antiox11091720)
Supplement: Supplementary file 1 [file antioxidants-11-01720-s001.zip › antioxidants-1852589-supplementary.pdf]

antioxidants

SUPPLEMENTARY MATERIAL

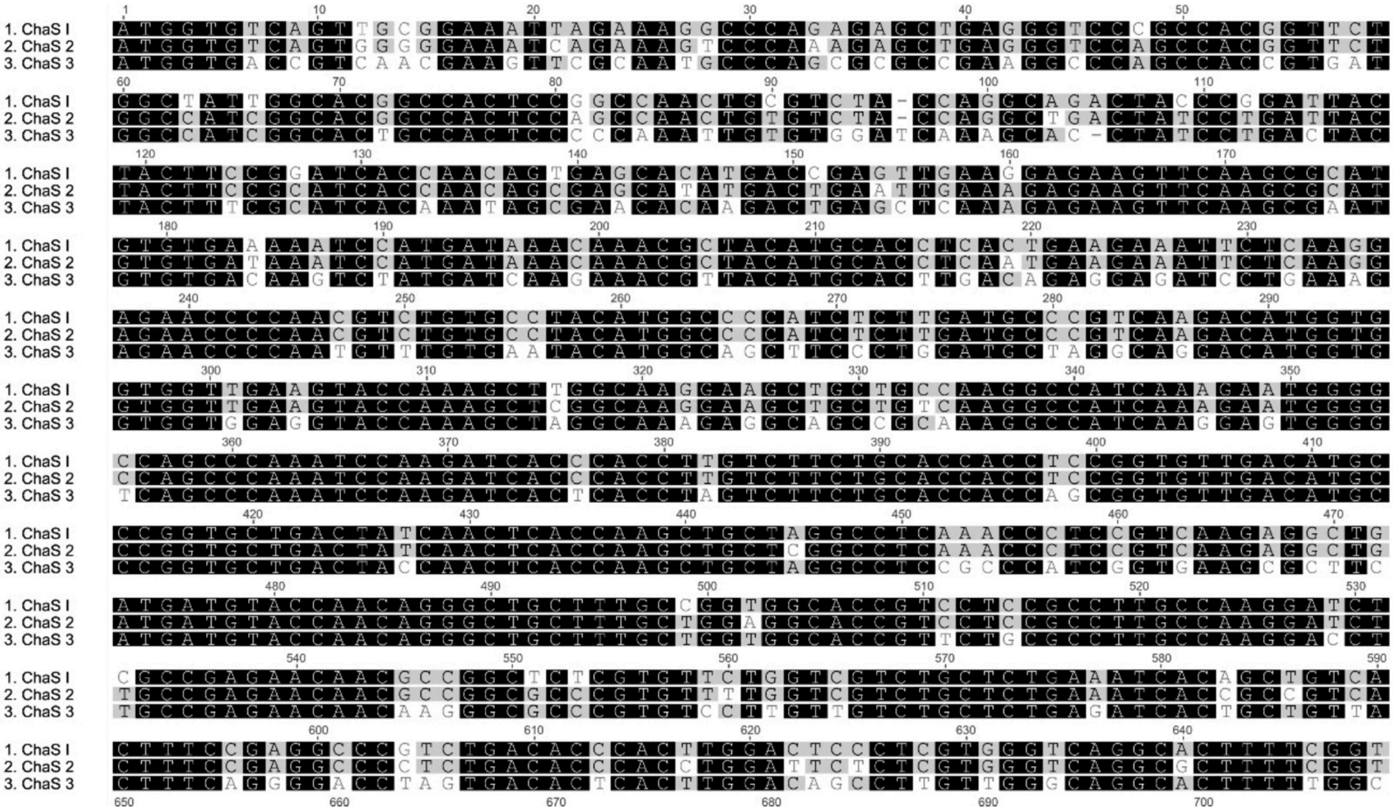

Figure S1. Nucleotide sequence alignment of the coding sequence of *ChaSI*, *ChaS2* and *ChaS3*.

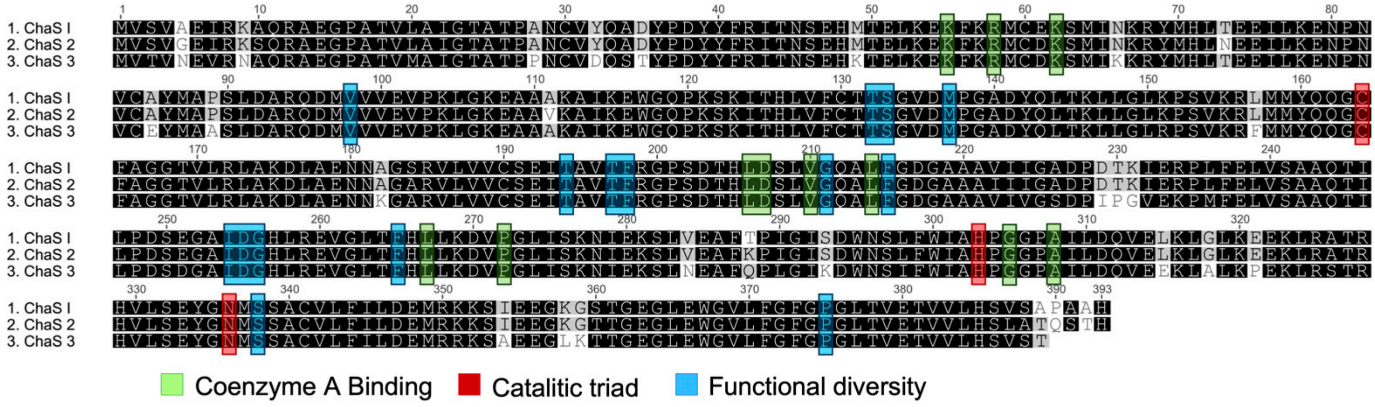

Figure S2. Alignment of the amino acid sequence of ChaSI, ChaS2 and ChaS3.

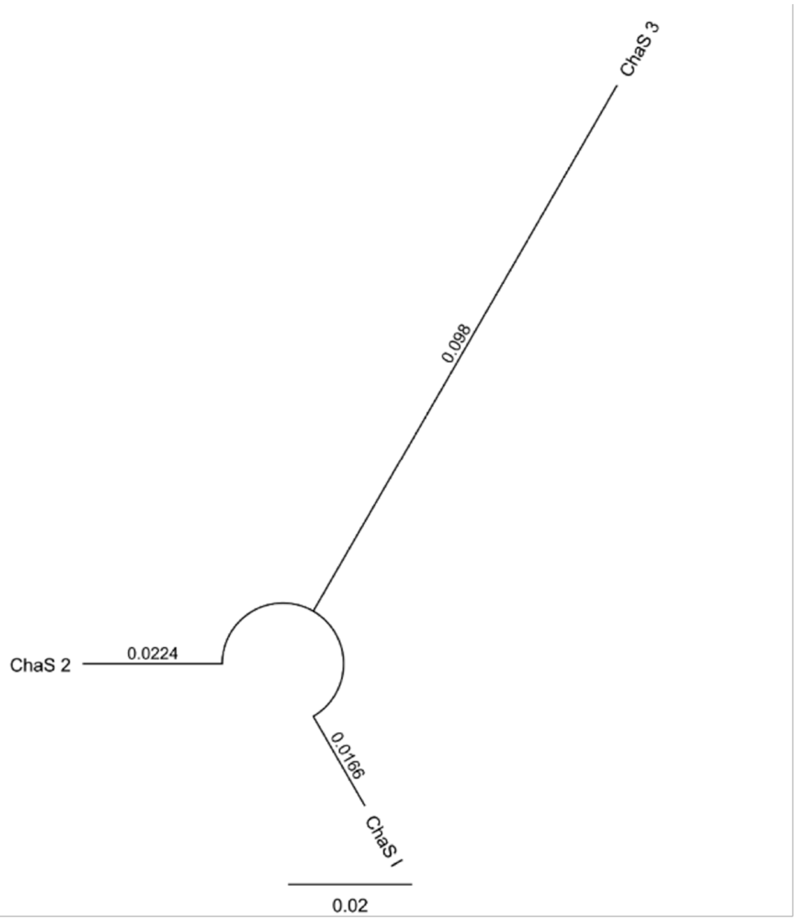

Figure S3. Phylogenetic tree from the amino acid sequence of ChaSI, ChaS2 and ChaS3.
